# Genome-wide identification, expression profile and evolutionary relationships of TPS genes in the neotropical fruit tree species *Psidium cattleyanum*

**DOI:** 10.1038/s41598-023-31061-5

**Published:** 2023-03-09

**Authors:** Drielli Canal, Frank Lino Guzman Escudero, Luiza Alves Mendes, Marcia Flores da Silva Ferreira, Andreia Carina Turchetto-Zolet

**Affiliations:** 1 Graduate Program in Genetics and Molecular Biology, Department of Genetics, Institute of Biosciences, Federal University of Rio Grande do Sul (UFRGS), Porto Alegre, Brazil; 2 Universidad Científica del Sur, Lima, Perú; 3 Departament of Chemistry, Universidade Federal de Viçosa, Viçosa, Brazil; 4 Laboratory of Genetics and Plant Breeding, Federal University of Espírito Santo, Alegre, Brazil

**Keywords:** Plant molecular biology, Secondary metabolism, Molecular evolution, Phylogenetics, Transcriptomics

## Abstract

Terpenoids are essential for plant growth, development, defense, and adaptation mechanisms. *Psidium cattleyanum* (Myrtaceae) is a fleshy fruit tree species endemics from Atlantic Forest, known for its pleasant fragrance and sweet taste, attributed to terpenoids in its leaves and fruits. In this study, we conducted genome-wide identification, evolutionary and expression analyses of the terpene synthase gene (TPS) family in *P. cattleyanum* red guava (var. *cattleyanum*), and yellow guava (var. *lucidum Hort.*) morphotypes. We identified 32 full-length TPS in red guava (RedTPS) and 30 in yellow guava (YlwTPS). We showed different expression patterns of TPS paralogous in the two morphotypes, suggesting the existence of distinct gene regulation mechanisms and their influence on the final essential oil content in both morphotypes. Moreover, the oil profile of red guava was dominated by 1,8-cineole and linalool and yellow guava was enriched in α-pinene, coincident in proportion to TPS-b1 genes, which encode enzymes that produce cyclic monoterpenes, suggesting a lineage-specific subfamily expansion of this family. Finally, we identified amino acid residues near the catalytic center and functional areas under positive selection. Our findings provide valuable insights into the terpene biosynthesis in a Neotropical Myrtaceae species and their potential involvement in adaptation mechanisms.

## Introduction

*Psidium cattleyanum* Sabine (Myrtaceae), commonly known as araçá, cattley guava, strawberry guava, and cherry guava, is a fleshy fruit belonging to the Neotropical Myrteae tribe (Myrtaceae). The species is native to the Atlantic Forest, where it has readily adapted to a variety of climates, is associated with wet forests across the tropics^1^, occurs in areas under stress conditions^2,3^, and is considered among the worst invasive species^4,5^.

The genus *Psidium* is rich in essential oils^6,7^, stored in the leaf secretory cavities^8–10^, and traditionally used for extraction, with inexpensive resources and potential uses in the pharmaceutical and medicine industries^2,11^. These essential oils regulate environmental processes and ecological interactions between organisms, such as defense against herbivores and pathogens^11,12^, protection against abiotic environments^13,14^ and attraction of pollinators, especially in neotropical species with fleshy berries that serve as a food source^15,16^.

*Psidium cattleyanum* species is divided into two morphotypes. The red guava (*P. cattleyanum Sabine var. cattleianum*) and yellow guava (*P. cattleyanum Sabine* var. *lucidum Hort*). The ripe fruits of red and yellow guava present red and yellow epicarps, respectively^17^. They also exhibit differences in antioxidant activity and phenolic content^18^, leaf morphology, and phytochemistry, size, and habit^19–21^. Previous studies have also detected considerably different oil profiles of yellow and red guava, which was attributed to differences in isolation techniques or the area of collection^22–25^. However, the genetic and evolutionary factors that can induce modifications in the secondary metabolism of the plant in the two morphotypes of *P. cattleyanum* remain largely unknown. Therefore, the study of genes of the biosynthetic pathway of these compounds in this species is highly relevant.

Because Myrtaceae species exhibit the highest concentrations and functional versatility of foliar terpenes among plants, significant efforts have been made to investigate the molecular mechanisms determining the structural diversity of terpene synthase (TPS) genes in this family. However, to the best of our knowledge, there are still no studies of these genes in Neotropical Myrtaceae species. The TPS family catalyzes the cyclization and rearrangement of geranyl diphosphate (GPP) or its cis-isomer neryl diphosphate (NPP) into monoterpenes (C10) and trans-geranyl diphosphate (GGPP) into diterpenes (C20) in the plastidic 2C-metil-D-eritritol-4-fosfato (MEP) pathway. In addition, farnesyl diphosphate (FPP) is converted into sesquiterpenes (C15) and triterpenes (C30) via the mevalonate (MVA) pathway in the cytosol, endoplasmic reticulum, and peroxisomes^26–29^. TPS controls not only the terpene chemodiversity present in plants but is also responsible for the unique composition of each taxon^30^.

Recent studies have revealed that, among those with dried capsular fruits, the species of the Eucalypteae tribe, including *Eucalyptus grandis*, *E. globulus,* and *Corymbia citriodora*, contain the largest number of complete TPS genes reported in eudicotyledons (70, 69, and 89 complete genes, respectively). This is due to the key role of terpenes in defense over their long lifespans^26,29,31^. Terpene synthase genes have also been identified in *Melaleuca alternifolia* and *Leptospermum scoparium,* with 37 and 49 putative TPS genes, respectively^32,33^. The oil profile patterns in foliar terpenes across this species, which are common in forest woodlands, are the monoterpenes α-pinene and 1,8-cineole. Instead, fleshy-fruited species from Myrtaceae family have low foliar 1,8-cineole concentrations, with a greater diversity of abundant foliar sesquiterpenes^34^.

In the present study, we aimed to conduct a comprehensive genome-wide analysis of TPS genes in *P. cattleyanum* to gain insights into the underlying mechanisms responsible for the differences in terpenoid biosynthesis and in the essential oil profiles in two morphotypes. Based on genomic and transcriptomic data, we identified the TPS gene repertoire and revealed its expression pattern in two *P. cathleyanum* morphotypes. We also examined the expansion and diversification of the TPS gene family among the Myrtaceae species. Finally, we investigated key amino acids using positive selection analysis to understand their effects on product specificity and consequently explain the chemical variability of the essential oil compounds. Our findings provide a foundation for deciphering TPS biosynthesis in *P. cattleyanum* and diversification of the two morphotypes. This knowledge will contribute to further studies on natural populations and the evolution of the Myrteae tribe, providing evidence of the successful distribution and adaptation of these species.

## Results

### Genome-wide identification of putative terpene synthases

We performed a genome-wide sequence homology search to identify the complete repertoire of TPS genes across the *Psidium cattleyanum* morphotype genomes. The genomes were assembled separately for comparison. Based on the conservation of hidden Markov model (HMM) profiles and BLAST searches, we identified 110 loci in the red genome (RedTPS) (Supplementary Table S1) and 106 loci in the yellow genome (YlwTPS) (Supplementary Table S2). Three RedTPS and seven YlwTPS sequences were excluded from further analysis due to the presence of premature stop codons, lack of both C and N terminal domains, presence of less than three exons, and one gene that presented 38 exons likely to be pseudogenes, partial genes, or assembly errors (Supplementary Table S3). In YlwTPS, 28 lost the C-terminal domain (PF03936), 45 lost the N-terminal domain (PF01397), and 33 of them contained two domains. In RedTPS, 27 lost the PF03936 domain, 49 lost the PF01397 domain, and 34 of them contained two domains (Supplementary Tables S1, S2). Of the remaining TPS gene models, only 32 RedTPS and 30 YlwTPS were classified as full-length putative loci coding genes (Supplementary Tables S1, S2). The number of TPS may be underrepresented due to incomplete sequences or atypical gene structures obtained and in part due to draft genome assembly.

To identify putative orthologs between the two morphotypes, we created a sequence percentage identity matrix (Supplementary Table S4), and genes containing the top hits are shown (Supplementary Table S5). Only four TPS partial genes had identical sequences in the two genomes (Pca_red_g91813 and Pca_ylw_g91315; Pca_red_g71488 and Pca_ylw_g60043; Pca_red_g21900 and Pca_ylw_g23854; Pca_red_g46186 and Pca_ylw_g20612). In addition, sequence identity among TPS genes between the two morphotypes was considerably lower, with only nine full length genes having greater than 90% amino acid identity (Supplementary Table S5). However, comparing partial and full genes, only 23 showed > 90% identity. The number increased when comparing only partial genes, where 29 genes showed > 90% identity (Supplementary Table S5).

**Figure 1:**
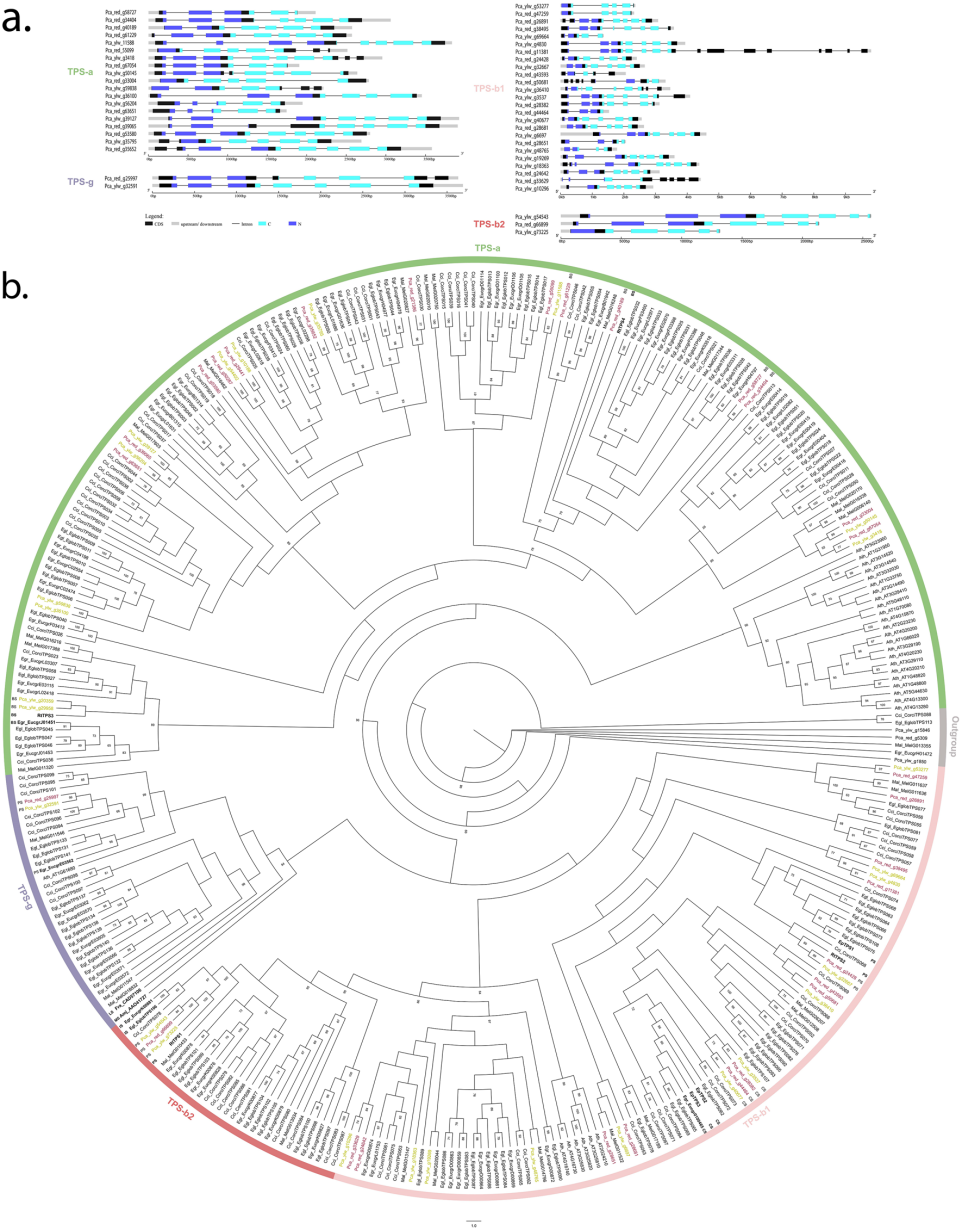
Phylogeny and gene structure of TPS from secondary metabolism. (**a**) Conserved domains in TPS genes and their consensus sequences from *P. cattleyanum.* (**b**) Phylogenetic tree of the Tps-a, Tps-b and Tps-g subfamilies from *P. cattleyanum* genome and characterized representative TPS from other Myrtaceae species. This tree was constructed through maximum likelihood analysis comparing the red and yellow morphotypes (Pca_red and Pca_ylw), *C. citriodora* subsp. variegata (Cci), *E. grandis* (Egr), *E. globulus* (Egl), *M. alternifolia* (Mal) and *A. thaliana* (Ath). Functional characterized terpene synthases are written in bold. Bootstrap values supported by < 60% are noted by number. A few species from TPS-c clade were used as the outgroup.

**Figure 2:**
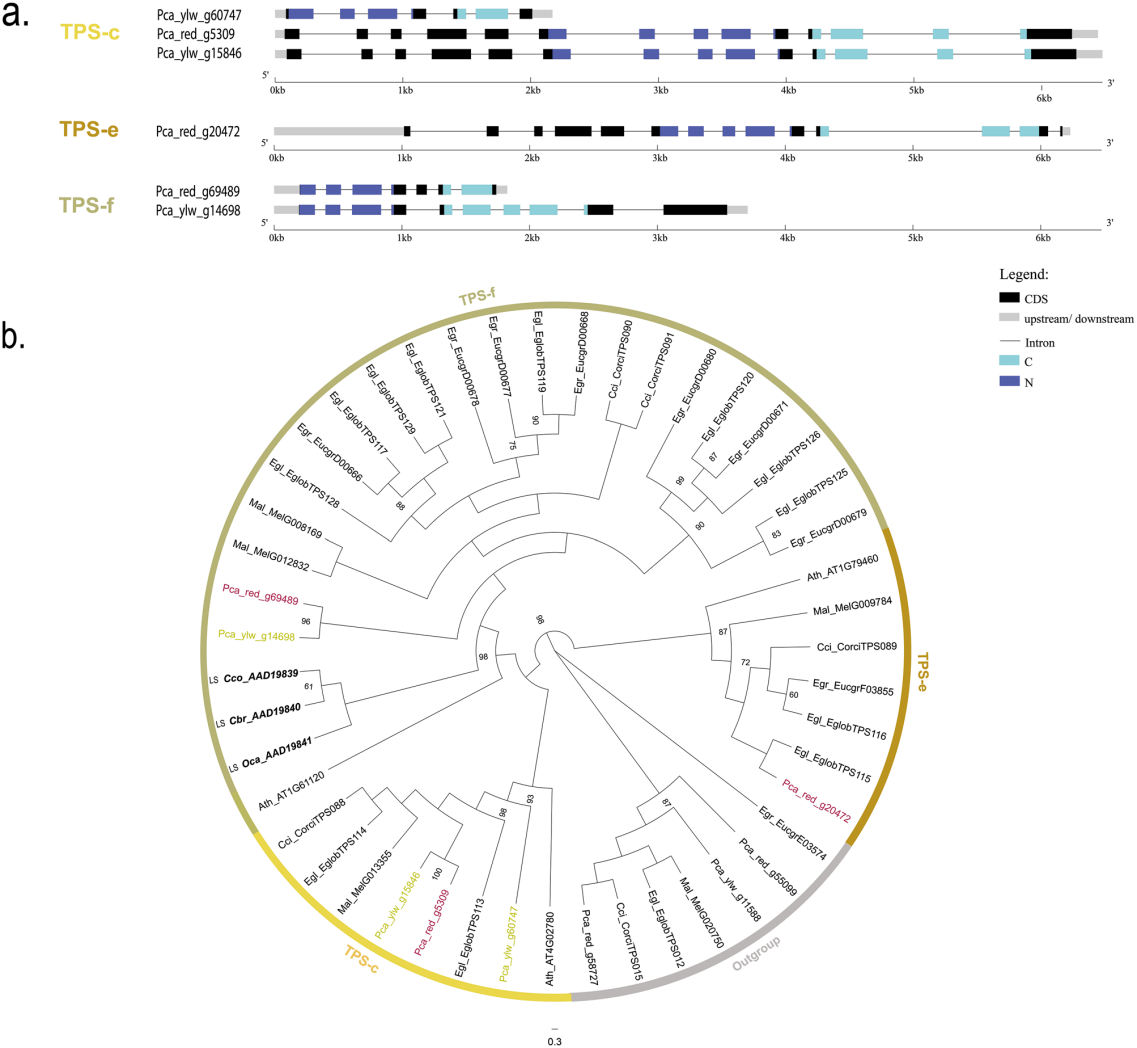
Phylogeny and gene structure of the TPS from primary metabolism. (**a**) Conserved domains in TPS genes and their consensus sequences from *P. cattleyanum.* (**b**) Phylogenetic tree of the Tps-c, Tps-e and Tps-f subfamilies from *P. cattleyanum* genome and representative TPS from other Myrtaceae species. This tree was constructed through maximum likelihood methods comparing the red and yellow morphotypes (Pca_red and Pca_ylw), *C. citriodora* subsp. variegata (Cci), *E. grandis* (Egr), *E. globulus* (Egl), *M. alternifolia* (Mal) and *A. thaliana* (Ath). Functional characterized terpene synthases are written in bold. Bootstrap values supported by > 60% are noted by number. A few species from TPS-a clade were used as the outgroup.

Most TPS genes of subfamilies TPS-a, TPS-b, and TPS-g contained six to nine exons (Fig. 1a), with exceptions (Supplementary Tables S1, S2). Genes from the remaining subfamilies, TPS-c, TPS-e, and TPS-f, contained 7–14 exons (Fig. 2A). Moreover, only one full YlwTPS (Pca_ylw_g56204) and four RedTTPS (Pca_red_g44464, Pca_red_g43593, Pca_red_g28651, and Pca_red_g25997) lacked the highly conserved aspartate-rich motif “DDXXD” (Supplementary Tables S1, S2). The TPS-c subfamily is present in land plants and is characterized by the “DXDD” motif but not the “DDXXD” motif in their proteins, which was detected in only one RedTPS and two full YlwTPS^26^. The second motif in the C-terminal domain, “NSE/DTE”, is less conserved in TPS and presents the variation “(L,I) × (D,N,G)D(F,I,L) × (S,T,G,A)xxxE”.

In the clade corresponding to the TPS-b subfamily monoterpene synthases, using different algorithm predictors, we found that only five full RedTPS and three full YlwTPS have an N-terminal transit peptide required for plastidial targeting (Supplementary Table S6).

We identified seven RedTPS and five YlwTPS with the N-terminal domain containing an “RRX8W" motif. In addition to these motifs, there is a highly conserved arginine-rich “RXR” motif. The TPS-g (Pca_ylw_g32591; Pca_red_g25997) subfamily is closely related to TPS-b; however, it lacks the conserved “R(R)X8W” motif in its encoded proteins, and its members may function in producing acyclic mono-, sesqui-, and diterpene products^26^.

### Molecular evolutionary analysis

To accurately classify the members of the *P. cattleyanum* TPS gene family based on sequence relatedness as well as functional assessments, we first collected 164 sequences of full-length TPS genes (containing the two TPS domains and having sequence lengths greater than 200 amino acids) from previous studies of species functionally characterized *A. thaliana* and *E. grandis *(Myrtaceae family) (Supplementary Fig. S2).

**Figure 3:**
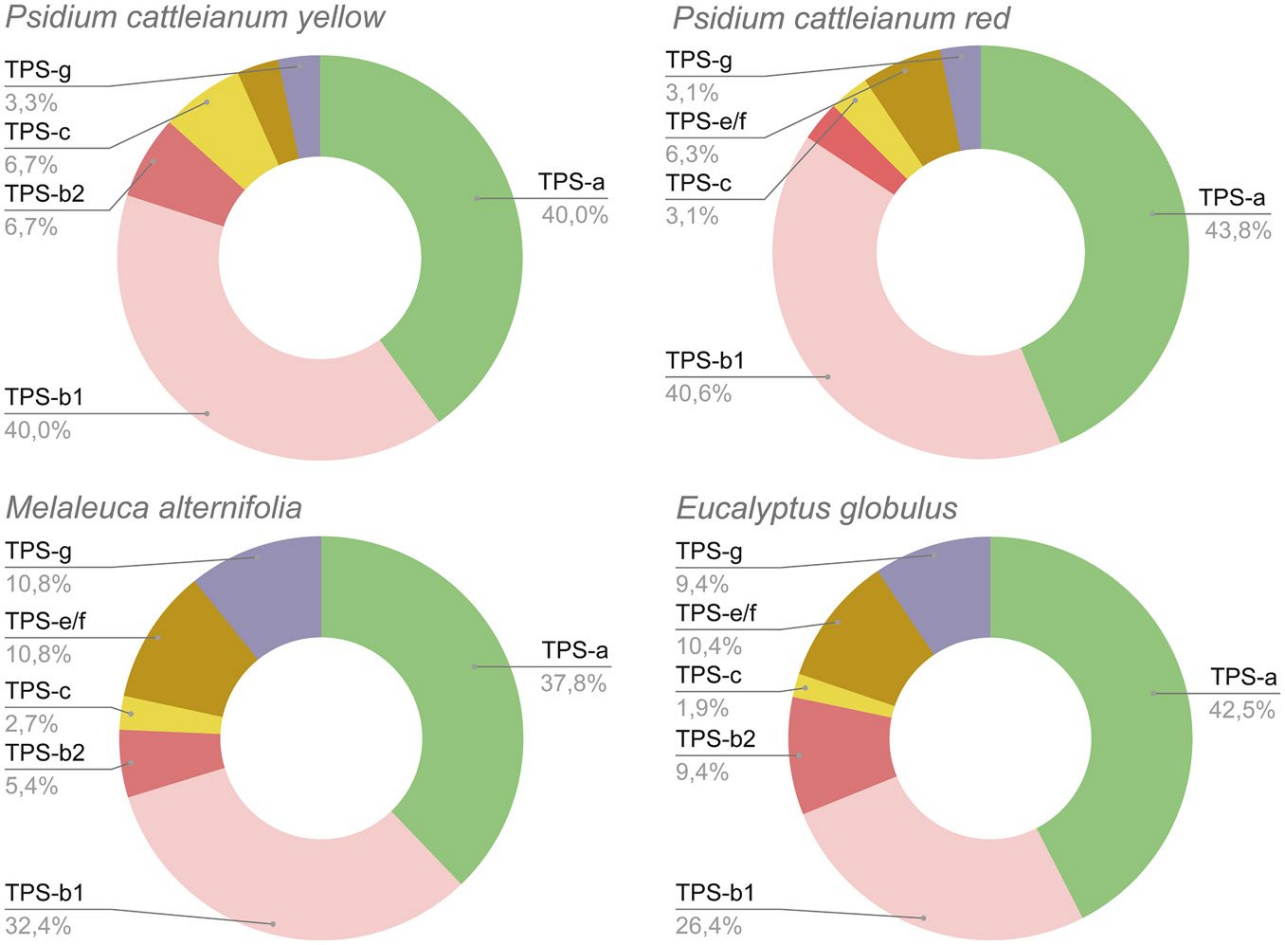
Proportion of TPS gene subfamilies found in Myrtaceae species. The number of genes in each subfamily relative to the total number of genes indicates the proportion of TPS genes. *Psidium cattleyanum* had the highest proportion of TPS-b1 genes (~ 40%).

The topology of the phylogenetic tree allowed us to divide TPSs into subfamilies belonging to secondary metabolism, clustered with subfamily TPS-a, which produces sesquiterpenes (C15) with 14 RedTPS and 12 YlwTPS (Table 1, Fig. 3), and TPS-b, which encodes enzymes that produce monoterpenes with 14 RedTPS and 14 YlwTPS. Only one TPS-g gene was found in each morphotype, which predominantly produced acyclic mono-, sesqui-, and diterpenes (Table 2, Fig. 1b). In the cluster representing primary metabolism, a single gene, TPS-c, which produces diterpenes (C20), was found in *P. cattleyanum* red morphotype, while two were found in the yellow morphotype (Table 2; Fig. 2B). In the TPS-e/f subfamily, which produces mono-, sesqui-, and diterpenes, a single gene was found in the yellow morphotype, whereas two were found in the red morphotype. Our analysis including other Myrtaceae TPS genes showed that all TPS proteins identified in this study clustered into monophyletic-specific clades related to the subfamilies. The TPS-a and TPS-b subfamilies were the most expanded, accounting for approximately 80% of the total TPS full length genes identified (Fig. 3).

**Table 1:** Chemical constituents of leaf oil from red and yellow morphotypes of *Psidium cattleyanum*.

N	Compound^a^	RI^b^	Content (%)^c^	Classification^d^	
Red	Yellow				
1	α-Pinene	930	10.0	35.4	MH
2	β-Pinene	972	–	3.2	MH
3	β–Myrcene	991	–	9.5	MH
4	1,8-Cineole	1028	59.5	22.4	MO
5	β-Ocimene	1039	2.9	–	MH
6	γ-Terpinene	1058	4.1	–	MH
7	Linalool	1100	9.6	3.7	MO
8	α-Terpineol	1189	5.6	2.7	MO
9	β-Caryophyllene	1414	2.5	2.7	SH
10	Nerolidol	1563	2.4	–	SO
11	Caryophyllene oxide	1579	–	6.6	SO
12	Viridiflorol	1598	–	2.2	SO
13	Aromadendrene epoxide	1633	–	2.5	SO
Total			96.6	90.9	

^a^Major compounds listed in the elution order using Rtx^®^-5MS column.

^b^RI: Retention index determined by the normalization of retention times with respect to an n-alkane mixture (C7–C40)^87–89^.

^c^Compounds with a relative area of > 2% were identified.

^d^Terpenic classification: oxygenated monoterpene (MO), hydrogenated sesquiterpene (SH), oxygenated sesquiterpene (SO).

**Table 2:** Numbers of TPS genes in Myrtaceae species.

Species	Total TPS gene models	Putative full length	Full length						
			a	b	c	d	e/f	g	h
*Psidium cattleyanum red*	110	32	14	14	1	0	2	1	0
*Psidium cattleyanum yellow*	106	30	12	14	2	0	1	1	0
*Corymbia citriodora*	127	84	38	33	1	0	3	9	0
*Melaleuca alternifolia*	37	37	14	14	1	0	4	4	0
*Eucalyptus globulus*	143	103	44	37	2	0	10	10	0
*Eucalyptus grandis*	172	70	38	15	1	0	8	8	0
*Leptospermum scoparium*	49	23	9	7	1	0	4	2	0

**Figure 4:**
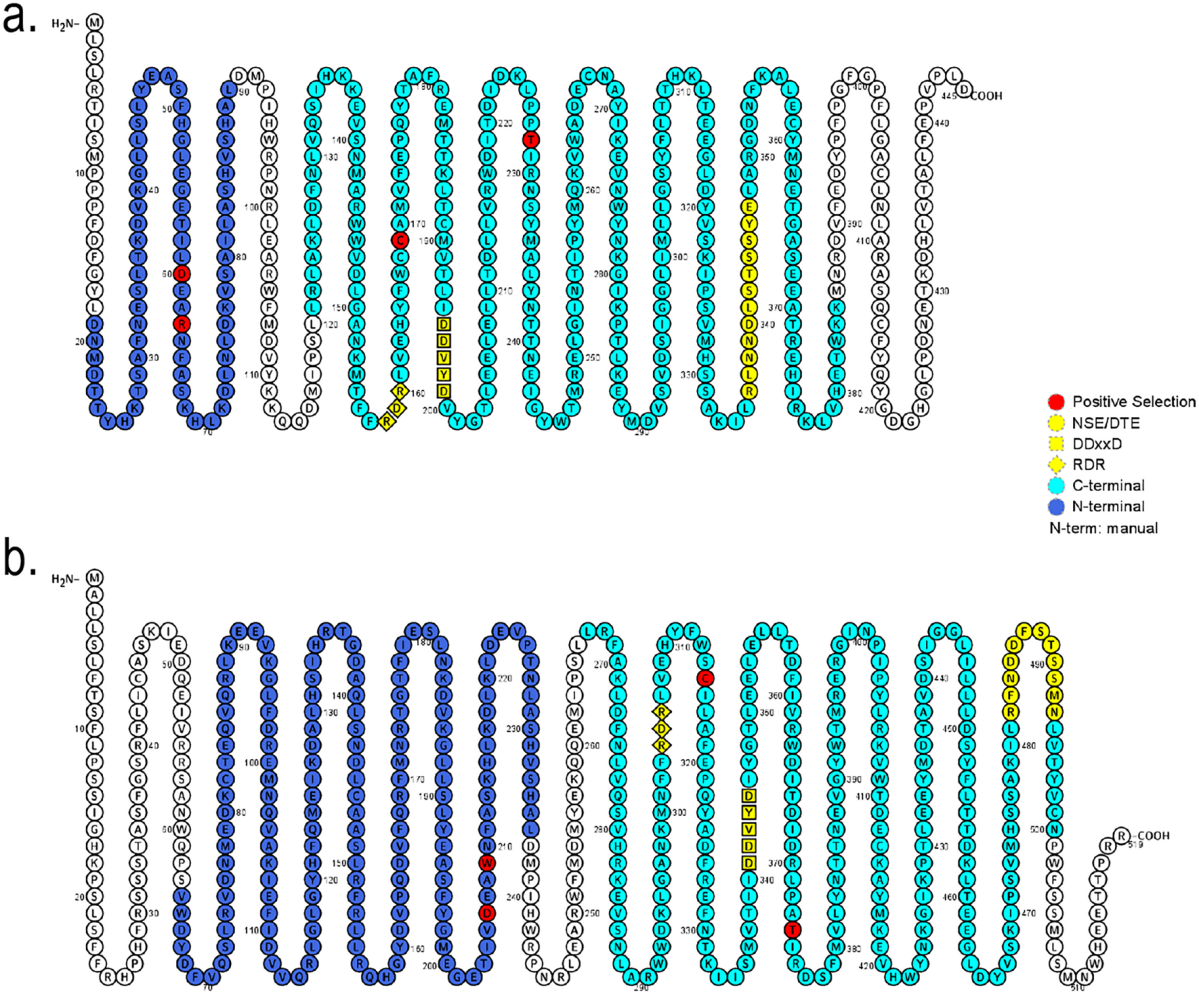
Positive selected sites in TPS-b1 branch including only *Psidium* genes. (**a**) The pinene synthase sequence Pca_red_g24428 representing clade 2 as foreground clade. (**b**) The linalool synthase sequence Pca_red_g28382 represents clade 2 as foreground clade. Amino acids that were identified on positive selection (red circles) are demonstrated on the protein sequence of these representative species corresponding to the sites in each alignment presented on Supplementary Table S12. Also, the representation of mainly motifs of the entrance of the active site (yellow square, circles, and triangle) represented for the “DDXXD”, “NSE/DTE” and “RXR” domains.

Site model selection analyses indicate sites that evolve under positive selection fit the data significantly better than the respective null models (M8 vs. M7: LRT = 14.46, df = 2, p = 0.001), however, the posterior probability was low (p < 0.55) (Supplementary Table S9). Therefore, positive selection may only occur during specific stages of evolution or in particular branches, we tested a branch-specific model to detect positive selection in the three clades formed in the TPS-b subfamily, which were fixed as foreground branches. Clade 1 contained only TPS-b1 genes from *Eucalyptus* and *Psidium* species. Clade 2 contained only TPSb-1 genes from *Psidium* species. A third clade contained some genes from *Populus*, *Vitis*, and *Eucalyptus* which were grouped with three pinene synthase genes from *Psidium* and classified as the only TPS-b2 genes. The one-ratio branch model indicated an overall purifying selection for TPS evolution (ω mean values smaller than 1.0). We also investigated selective pressure using the branch site model according to the likelihood ratio tests (LRT) and comparisons of clade 1 (p = 0.26), clade 2 (p < 0.05), and clade 3 (p < 0.05), indicating that some sites were statistically significant (Supplementary Table S12). However, only five residues were strongly identified to be under positive selection in clade 2, located in the N-terminal portion of TPSb-1 genes of *Psidium*, including residue 121, with an aspartate (D) and the alteration to a leucine (L) in the foreground branches, and residue 124 with the most commonly found lysine (K), arginine (R), or tryptofan (W) and its alteration to alanine (A) in foreground branches. We also detected residues 222 with a cysteine (C) and alteration to valine (V) or leucine (L) in the foreground branches, and site 279 with a threonine (T) or isoleucine (I) that presented an alteration to cysteine (C) or tyrosine (Y) in the foreground branches, around “RDR” and “DDXXD” motifs in the C-terminal portion (Fig. 4). Clades 1 and 3 show the residuals with weak signs of positive selection.

### Global and differential expression analysis associated with the terpene biosynthesis

To gain more insight into the TPS biosynthetic pathway, global and differential expression profiles were evaluated on TPS genes from RNA samples extracted from its leaves and compared in two morphotypes. After library construction, illumina sequencing, and assembly, approximately 84 and 86 million paired end reads already cleaned were generated for yellow and red morphotypes, respectively.

Looking at total gene expression across the two morphotypes, approximately 30% of TPS genes were expressed in leaves (transcripts anchored in 35 genes in the red genome and transcripts anchored in 30 genes in the yellow genome). Genes that showed some expression patterns fell into five clades (Supplementary Tables S7, S8). We found 17 full-length and 18 partial TPS genes with evidence of expression in the red genome and 13 full-length and 18 partial TPS genes in the yellow genome.

**Figure 5:**
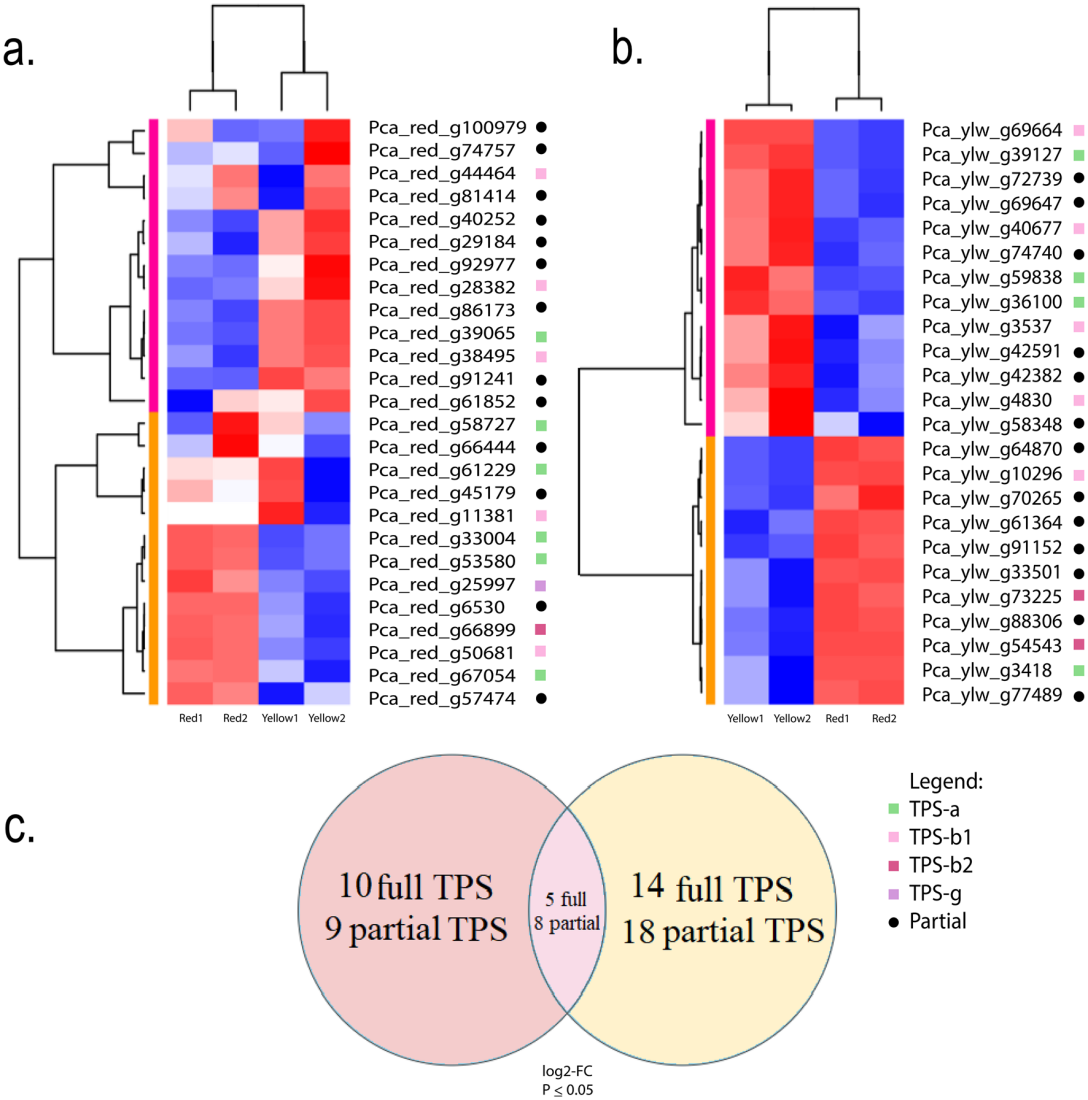
Differential expression of terpene synthase genes of *P. cattleyanum* leaves. (**a**) Red morphotype genome. (**b**) Yellow morphotype genome. The blue colored cells indicate the Log2 transformed FPKM (unit of fragments per kb of exon per million mapped reads) with no expression and value zero in this tissue (down-regulated), and red color indicates a higher percentage of total expression for a given gene (up-regulated). Squares represent full-length genes and black dots represent partial genes. (**c**) Veen Diagram representing the up-regulated unique transcripts common between the morphotypes.

A heat map showing differential gene expression using DESeq2 based on |log2Fold Change |≥ 1 and FDR < 0.05 in the red and yellow morphotypes, with two biological replicates in leaves, is shown in Fig. 4. As the two genomes were assembled separately and belonged to the same species, two heatmaps were generated, anchoring all transcripts in the red genome (Fig. 5A) and all transcripts in the yellow genome (Fig. 5B). Therefore, statistical analysis can be performed and then compared. Among these, 19 gene sequences were upregulated in the red morphotype, with only 10 full TPS genes (Fig. 5C; Supplementary Tables S7, S8). In the yellow morphotype, 32 TPS genes were upregulated, but only 14 were full TPS. A total of 12 TPS genes showed the same expression pattern between the two transcriptome comparisons and > 90% of identity, indicating that the same gene was found in the different genome assemblies.

### Terpenoid profling in *Psidium cattleyanum* leaves

The leaves of *Psidium cattleyanum* were examined for chemical compositions of the volatile terpene compounds, to investigate the genetic influence on the chemical variations of the oil content between the two morphotypes*.* The content of each terpenoid was calculated as a percentage of the total essential oil using gas chromatography with a flame ionization detector (GC-FID) and gas chromatography coupled to mass spectrometry (GC–MS) approaches. Thirteen compounds were identified, and the most abundant monoterpenes in both morphotypes were 1,8-cineole, α-pinene, linalool, and α-terpineol (Table 1; Supplementary Fig. S1A). Although these compounds were commonly found, they showed significant quantitative variation. For example, the α-pinene showed a large difference of 35.4% in yellow and only 10.0% in red morphotype; 1,8-cineole showed a difference of 59.5% in the red and 22.4% in yellow morphotype; whereas linalool showed a difference of 9.6% and 3.7% in the red and yellow morphotypes, respectively.

In addition to quantitative variations, the plants used also showed qualitative variations in the chemical composition of their essential oils. The hydrogenated monoterpenes β-ocimene (2.9%) and γ-terpinene (4.1%) were observed only in red morphotype essential oil, and the oxygenated sesquiterpene nerolidol (2.4%) (Supplementary Fig. S1B). The hydrogenated monoterpenes β-pinene (3.2%) and β-myrcene (9.5%) were observed only in yellow morphotype, and the oxygenated sesquiterpenes caryophyllene oxide (6.6%), aromadendrene epoxide (2.5%), and viridiflorol (2.2%) (Supplementary Fig. S1C).

## Discussion

The Myrtaceae family is recognized for its great potential to produce volatile oils of economic interest^35^. The identification of photochemical profiles of some species combined with genomic studies, revealing a high diversity of TPS genes that control the synthesis pathways of these compounds and are responsible for the various biological activities of essential oils^28,29,31,36^.

In this study, the TPS family has been characterized in *Psidium cattleyanum*, a fleshy-fruited species from the Myrtaceae family, for the first time at the genomic and transcriptomic levels. It reveals a low number of putative functional full-length TPS genes (32 RedTPS and 30 YlwTPS) required for this species associated with wet forests across the neotropics, when compared with the woody-fruited species (Table 2) from open forest and woodland, such as Eucalypteae tribe, including *Eucalyptus grandis* (70 full length TPS), *Eucalyptus globulus* (103 full length TPS), and *Corymbia citriodora* (84 full length TPS), all species with the diversity center in the Asia and Oceania^37^. These species are predicted to defend their leaves much more strongly. Moreover, the relatively long lifespan of eucalyptus (well over 200 years)^33^ compared to *Psidium* (approximately 40 years)^38^, may drive further gene diversification as the need to adapt to long-term environmental changes. These results imply that evolutionary forces have acted differently upon lineages since they diverged from their most recent common ancestor more than 70 million years ago^1,39^.

Partial genes might be considered non-functional, even though some of their incomplete sequences could have resulted from poor sequencing techniques. Still, the redundancy of TPS genes has been observed in many other plants, e.g., in grape (*Vitis vinifera*) there are 152 TPS-like genes, but only 62 full length TPS, with two domain structures^40^ where tandem duplication rates for both domains (~ 90%) are the main mechanisms for family expansion. In *E. grandis* there were 70 full-length TPS, but seven had only the PF01397 domain where gene losses were mostly related to tandem duplications (71.4%) and less related to segmental duplication (3.9%) events, and 22 TPS with only the PF03936 domain more related to tandem duplication (71.7%) and fewer segmental duplication events (4.3%)^41^. We observed the same pattern in *Psidium cattleyanum*, where 28 RedTPS and 27 YlwTPS had only the PF01397 domain and 48 RedTPS and 45 YlwTPS had only the PF03936 domain (Supplementary Table S9). These data suggest that domain loss has been a common event in plants during the evolution of the TPS gene family, with the loss of the PF01397 domain being more frequent in the Myrtaceae family and plants in general than the loss of the PF03936 domain^41^. The functionality of these single domain-containing TPS is not yet known, but more investigation on regulatory mechanisms, expansion history, and evolutionary advantage of the domains separately should provide a comprehensive view of the impact of partial genes in the diversification of TPS in plants^26^.

Transcriptome examination revealed that out of 32 full-length RedTPS, 10 genes were upregulated in the red morphotype. Among the 30 full-length YlwTPS, only 14 genes were upregulated in the yellow morphotype. This demonstrates that the differential expression patterns in the two morphotypes can also contribute to the final terpene content in the leaves (Fig. 5). The high abundance of transcripts in this study (FPKM, Fragments Per Kilobase of exon per Million reads) from the TPS-a and TPS-b1 subfamilies in the transcriptome indicated their involvement in the formation of mono and sesquiterpenoid volatiles in leaves.

A comparison of the essential oil composition revealed the presence of oxygenated monoterpenes on leaves of *P. cattleyanum*, where the major compound was α-pinene (35.4%) in yellow morphotype and the 1,8-cineole (59,5%) and linalool (9.6%) in the red morphotype. This variation in the essential oil of *P. cattleyanum* morphotypes have also been previously described in native species in southern Brazil^24,25^. In cultivated plants of *P. cattleyanum* in different parts of the world, previous studies have identified the chemical composition with β-caryophyllene, a hydrocarbon sesquiterpene, as the main component^7,23,25,42–45^, which was also found in smaller amounts in both morphotypes in this work.

The variations found between the two morphotypes in this study reflect a genetic and evolutionary origin. The identification of chemotype phenotypes (qualitative variability in foliar essential oil composition) within a single species has already been reported among different varieties or ecotypes of other species^46–49^, mainly when a significant shift in the relative concentrations involved more similar compounds, such as cineole and pinene^50^. The yellow morphotype tends to be found at slightly lower elevations than the red morphotype^17,21^, this could reflect environmental adaptation^48^ and also in the terpenes plasticity^51^.

**Figure 6:**
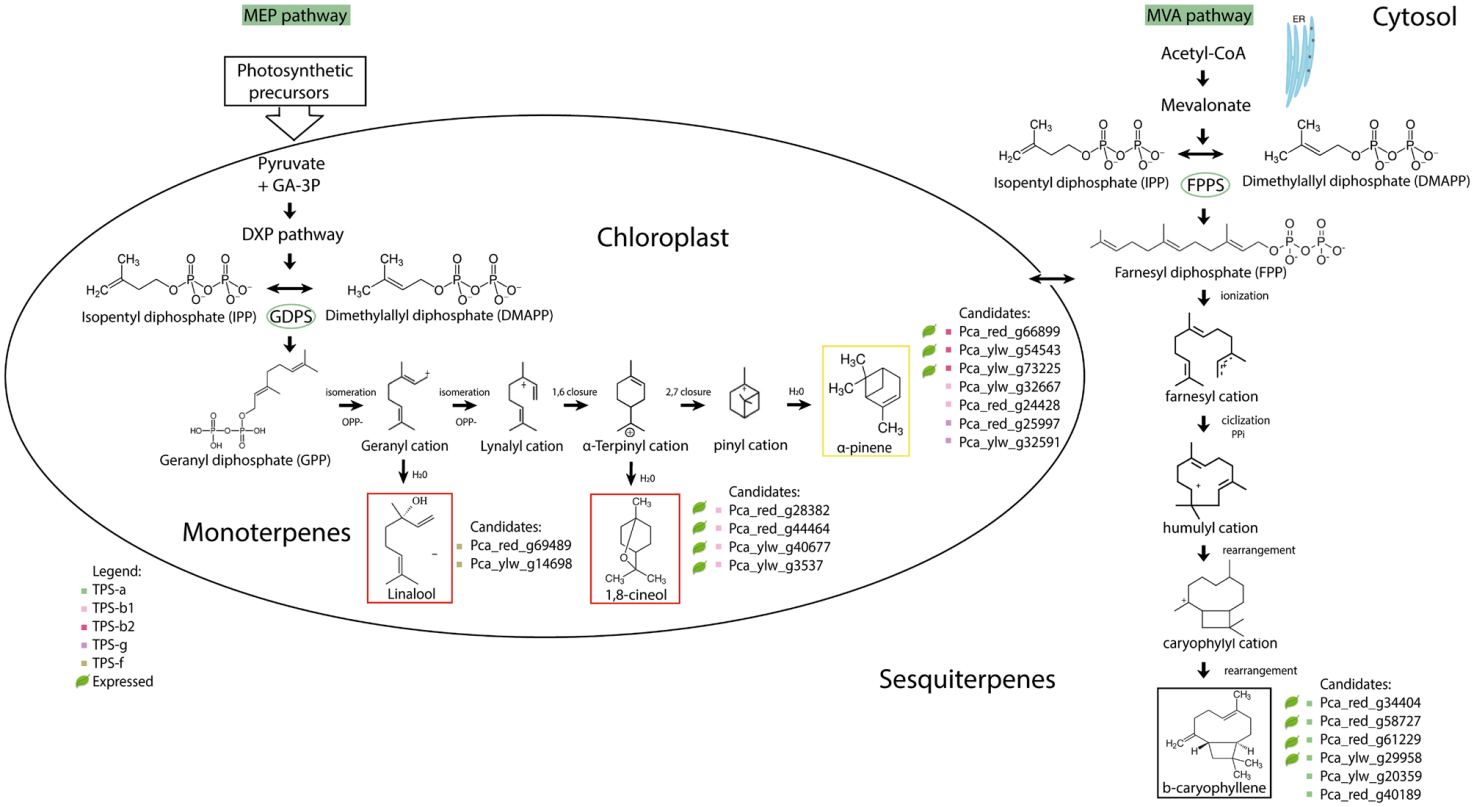
A schematic view of putative terpene synthase genes involved in α-pinene, 1,8-cineol, linalool and β-caryophyllene biosynthesis in *P. cattleyanum* red and yellow morphotypes. *DXP* 1-deoxylulose 5-phosphate, *DMAPP* dimethylallyl diphosphate, *IPP* isopentenil diphosphate, *GPPS* geranyl diphosphate synthase, *GPP* geranyl diphosphate, *FPPS* farnesyl diphosphate synthase, *FPP* farnesyl diphosphate, *ER* endoplasmic reticulum, *MEP* methyl erythritol 4-phosphate pathway, *MVA* mevalonate pathway.

In the TPS-a subfamily that encodes only sesqui-TPSs found in both eudicot and monocot plants^40^, phylogenetic analysis revealed two YlwTPS (Pca_ylw_g29958 and Pca_ylw_g20359) closely related to RtTPS3 (AXY92168)^52^ and in the same branch of the gene EgranTPS038 (Euc_Eucgr_J01451) of *E. grandis*. In addition, four RedTPS (Pca_red_g40189, Pca_red_g58727, Pca_red_g34404, and Pca_red_g61229) were found in the same branch as RtTPS4 (AXY92169)^52^; both belong to a branch of the betacaryophylene synthase (BS) (Fig. 6).

The monophyletic TPS-b subfamily is divided into two groups. The TPS-b1 clade contains putative cyclic monoterpene synthases, with transit peptides positioned upstream of the “RRX8W” motif and therefore has a high probability of localizing in the plastids^29^. The subfamily had the highest number of full-length genes (40%) as a proportion of the total number of TPS genes compared to *Melaleuca alternifolia* (32.4%) and *Populus trichocarpa* (31.2%) (Fig. 3). The high proportions of the TPS-b1 subfamily could be indicative of rapid ongoing evolution and lineage-specific gene family expansion of this subfamily in warm subtropical habitats, particularly for protection from damage caused by rapid temperature fluctuations^53,54^. Some terpenes can act by selecting the defense of antimicrobial secondary metabolites such as cyclic monoterpenes^32^. This suggests that subfamilies of TPS-b1 expansion might be related to species or ecotype diversification, enabling quick adaptation in response to environmental changes.

The other TPS-b subfamily contains putative isoprene/ocimene (C5, C10) synthases, described as TPS-b2^32^ and has few genes in P*sidium cattleyanum* (6.6% in yellow and 3.1% in red morphotype) as a proportion of the total number of TPS genes compared with *Eucalyptus globulus* (9.4%) and *Melaleuca alternifolia* (5.4%) (Fig. 3)^29,32,55^. However, when including genes functionally characterized in the TPS-b2 clade, the relationships among *Psidium* genes were not entirely congruent because we detected these three genes (Pca_ylw_g54543, Pca_red_g66899, and Pca_ylw_g73225) positioned in the same clade as *Rhodomyrtus tomentosa* RtTPS1 (AXY92166), characterized as pinene synthase (PS), a cyclic terpene^52^, despite the high support (bootstrap value of 97) in the same branch of acyclic EglobTPS106, functionally characterized as isoprene synthase^29^ (Fig. 1b). Including more TPS from Myrtaceae species from neotropics and functionally characterizing the *Psidium* genes should clarify their role and division within the clade TPS-b.

Two other genes (Pca_ylw_g32667 and Pca_red_g24428) clustered together with RtTPS2 (AXY92167) and EpTPS1 (MK873024) in the TPS-b1 branch and were related to pinene synthase (Fig. 6). Moreover, four genes (Pca_red_g44464, Pca_red_g28382; Pca_ylw_g3537, Pca_ylw_g40677) were in the same branch as EpTPS2 and EpTPS3 from *Eucalyptus polybractea* belonging to the CS TPS-b1 clade^56^, and one gene from *E. grandis* monoterpene synthase (XP_010046521), which has similarity (95% amino acid identity) to CS that produces 1,8-cineole an in vitro assay using GPP as substrate^57^. Analysis of transcript abundance showed that the gene Pca_red_g28382 was highly expressed in leaves. The dominant product of many characterized CS enzymes is 1,8-cineole; however, they also produce small amounts of limonene, β-myrcene, sabinene, β-pinene, α-pinene, and α-terpineol. This group of compounds synthesized by CS is known as the ‘cineole cassette’, which has been reported in many plants^58,59^. Therefore, as multiple TPS genes are often expressed in the same tissue and many of these TPS’ have overlapping ranges of products, it is not easy to identify the action of individual TPS enzymes on the profile of the terpene observed in that tissue^60^.

Other expressed TPS-b genes Pca_ylw_g54543, Pca_red_g66899, and Pca_ylw_g73225 were positioned in the same clade as RtTPS1 of *Rhodomyrtus tomentosa*, and Pca_red_g24428 grouped with RtTPS2. Previous studies have shown the in vitro activity of RtTPS1 and RtTPS2, which mainly produce (+)-α-pinene and (+)-β-pinene with GPP, whereas RtTPS1 is also active with FPP, producing β-caryophyllene, along with a smaller amount of α-humulene^53^. This suggests that, depending on their expression profile or subcellular location, the enzymatic products of these TPS present in leaves can contribute to the different terpene mixtures found in the essential oil. We also detected the expression of four genes (Pca_red_g44464, Pca_red_g28382; Pca_ylw_g3537, Pca_ylw_g40677) in the same branch of EpTPS2 and EpTPS3, belonging to the PS TPS-b clade.

There is a large diversity of Myrtaceae species, with α-pinene and 1,8-cineole being the dominant compounds in the leaves. The reaction cascade that leads to these two compounds includes the same carbocation intermediate, γ-terpinyl cation^49^. There is evidence to show that the amino acid changes induced through site-directed mutagenesis can result in a different ratio of particular terpenes produced^47,61,62^ and in natural systems, this might lead to different dominant compounds, such as the a-terpineol synthases of many species, which are the only characterized terpene synthases that are not 1,8-cineole synthases, but produce significant amounts of 1,8-cineole^59^.

The TPS-g subfamily has two subclades encoding TPS’ without the “R(R)X8W” motif, which facilitates isomerization of the geranyl cation in the linalyl cation. This subfamily is closely related to the TPS-b subfamily, and its members may function with the prevalence of acyclic monoterpene products. We also identified two genes from *Psidium* (Pca_red_g25997 and Pca_ylw_g32591) in the same branch as the functionally characterized PS of EgranTPS101^29^ (Egr_EucgrE03562; Fig. 6).

We screened TPS genes to identify the LS based on functionally characterized enzymes from other plant species. Phylogenetic analysis demonstrated that only two genes (Pca_ylw_g14698 and Pca_red_g69489) are closely related to LS from the rosids *Clarkia breweri* (Cbr_AAD1984)*, Oenothera arizonica* (Oca_AAD1984)*,* and *Clarkia concinna* (Cco_AAD1983). They fall into the TPS-f synthase classification, proposed to be the most ancient, and could have been due to a relatively recent common ancestor, copalyl diphosphate synthase (CPS)^63^, as evidenced by the sequence conservation of this region in the N-terminus of the protein (Fig. 6). In this study, LS gene expression was not observed in leaves. Monoterpene synthases of this subfamily are responsible for the conversion of GDP into the bulk of monoterpenes found in vegetative organs, whereas the subfamilies TPS-f and TPS-g are thought to be exclusively active in flowers, likely having a primary function in attracting insect pollinators^36,64^. In addition, other genes could be expressed when directly involved in plant defense against herbivores by attracting predators^65^ or by directly driving herbivores away^66^.

Depending on the extent to which gene function is affected, single-base substitutions may result in changes in terpene composition and profile, and if upstream pathway elements are involved, even in terpene concentrations^46,67^. To infer whether selection acted on the TPS-b subfamily, we used several statistical tests to compare clades on the phylogenetic tree. Codon substitution patterns with a maximum likelihood approach implementing a branch-site model indicated positive selection acting on a specific TPS-b1 branch, including some pinene and cineole synthase genes and other non-functionally assigned genes.

In particular, some positively selected sites are located in the N-terminal region, which controls substrate specificity. It is interesting to note that residue 224 contains an arginine (R) in the PS genes (Fig. 4A), whereas we observed an alteration to a tryptophan (W) residue in the CS genes (Fig. 4B). Conserved arginines close to the diphosphate moiety stabilize the evolving negative charges^68^. The tryptophan residue contributes to stabilization of the cation and deprotonation of the substrate^69^. In addition, the positively selected residues 222 and 279 were located around the aspartate-rich motif (“DDXXD”) in the C-terminal half, which is important for the coordination of divalent ion(s), water molecules, and stabilization of the active site^70–72^.

These results illustrate the importance of these residues to product spectrum of TPS genes, mainly in this case of PS and LS, that have the same carbocation intermediate, thereby differing in their profiles^46^. Future studies should investigate in detail how the active site promotes discrimination from other potential substrates. Analysis of this type of data could be used to better understand the diversity of terpene synthases and the role of different terpenes in mediating ecological interactions^34^.

Several biological and pharmacological activities have been reported for pinene, cineol, and linalool, including anti-inflammatory and antinociceptive properties^11,73–75^, anticancer^61,76,77^, antifungal^78,79^, antidiabetic^80^, antioxidant, antimicrobial^77,81,82^, antidepressive and neuroprotective^77^, allelopathic^83^, antibacterial, and insecticidal activity^84,85^. The high content of these compounds in the volatile oils of these species suggests that they could constitute an alternative commercial source of this compound^86^.

## Conclusion

In this study we identified putative TPS genes responsible for the formation of predominant essential oil compounds in *Psidium cattleyanum*. The chemotypic variability found in the red and yellow morphotypes confirm our hypothesis about the complex and polymorphic nature of the genes encoding the key enzymes regulating compound production and suggest adaptive genetic plasticity of the two morphotypes. The TPS-b clade has undergone substantial expansion compared to other subfamilies and includes some positively selected amino acid residues, evidence the monoterpene synthase genes are important for adaptation to *Psidium* at different niches. The present study provides the first insight into the genetic basis of TPS in *P. cattleyanum* morphotypes, gaining insights about the biodiversity in the Atlantic rainforest for further ecological genetic studies in the genus.

## Materials and methods

### Plant materials

Young leaf samples of the yellow and red morphotypes were grown on the same open ground plot (in two 5-m long rows per cultivar) at the Federal University of Rio Grande do Sul (Porto Alegre, Brazil). The plants were 20–25 years old during the sampling year (2020). The leaves were washed with distilled water, frozen, and stored at -18 °C until extraction of volatile compounds, immediately frozen in liquid nitrogen and stored at − 80 °C for further RNA extraction.

### Chromatographic profile of the essential oils

We collected volatiles from the leaves of the two morphotypes under the same growth conditions and ambient temperature, in biological triplicates. Approximately 100 g of dry leaves from the two morphotypes, were extracted with 1000 mL of reverse osmosis water using a Clevenger apparatus^87^, following four hours of extraction by hydro-distillation. Samples of the essential oils extracted from the leaves were analyzed using gas chromatography with a flame ionization detector (GC-FID) (Shimadzu GC-2010 Plus) and gas chromatography coupled to mass spectrometry (GC–MS) (Shimadzu GCMS-QP2010 SE).

We conducted the analyses according to the following conditions: helium (He) as the carrier gas for both detectors, with the flow and linear speeds of 2.80 mL min^−1^ and 50.8 cm s^−1^ (GC-FID), and 1.98 mL min^−1^ and 50.9 cm s^−1^ (GC–MS), respectively; injection port temperature of 220 °C with a split ratio of 1:30; fused silica capillary column (30 m × 0.25 mm); stationary phase Rtx^®^-5MS (0.25 μm film thickness); oven with an initial temperature of 40 °C, maintained for 3 min, then gradually increased by 3 °C min^−1^ until 180 °C, where it remained for 10 min (total analysis time: 59.67 min); and FID and MS detector temperature of 240 °C and 200 °C, respectively^49^. The used samples were taken from the vials in 1 μL of a solution containing 3% essential oil dissolved in hexane with 0.1 mol L^−1^ dimethylacetamide (DMA; external standard for reproducibility control).

The GC–MS analyses were performed using electron impact equipment with an impact energy of 70 eV, scanning speed of 1000, scanning interval of 0.50 fragments s^−1^, and fragments detected from 29 to 400 (*m/z*). The GC-FID analyses were carried out in a flame formed by H2 and atmospheric air at a temperature of 300 °C. Flow rates of 40 mL min^−1^ and 400 mL min^−1^ were used for H2 and air, respectively. Identification of the compounds in the essential oils was accomplished by comparing the obtained mass spectra with those available in the spectral library database (Wiley 7, NIST 05, and NIST 05 s) and retention indices (RI). To calculate the RIs, we used a mixture of saturated alkanes C7–C40 (Supelco-USA) and adjusted retention time of each compound, obtained by GC-FID. The values calculated for each compound were compared with those reported in literature^88–90^.

We calculated the relative percentage of each compound in the essential oil using the ratio between the integral area of the peaks and the total area of all sample constituents obtained via GC-FID analyses. The compounds with a relative area above 2% were identified and considered predominant if above 10%.

### Terpene synthase gene identification and annotation

Initially, we used two terpene synthase-specific domains, PF01397 and PF03936, which represent respectively the N-terminal and C-terminal domains of TPS from the Pfam database (http://pfam.xfam.org/) ^91^, as queries to search for terpene synthase homolog genes in the *P. cattleyanum* yellow and red morphotypes predicted genes from their genomes (unpublished data). We analysed each morphotype separately using HMMER version 3.1^92^. We also performed a local BLASTP search for TPS genes in the *P. cattleyanum* reference genome based on functionally characterized genes^93,94^. We created a preliminary list of putative TPS genes based on hits with a high similarity (e-value < 1e − 05).

To better understand the structural sequence features of each gene, we used the open reading frame (ORF) Finder of NCBI (http://www.ncbi.nlm.nih.gov/orffinder/) to identify the ORFs for each sequence recovered. Gene structure was determined using the Gene Structure Display Server (GSDS; http://gsds.cbi.pku.edu.cn) ^95^. We confirmed the presence of functional domains based on the translation of gene sequences identified in Simple Modular Architecture Research Tool (SMART)^96^. Moreover, several algorithms were used to predict a putative transit peptide for chloroplast targeting in the N-terminal sequence upstream of the RRX8W motif (ChloroP 1.1^97^, TargetP v.1.01^98^, PCLR 0.9^99^). To determine the sequence diversity between the two morphotypes, a complete set of pairwise comparisons of protein sequences was performed using Clustal Omega (https://www.ebi.ac.uk/Tools/msa/clustalo/).

### Phylogenetic reconstruction

In this study, we first used terpene synthase protein sequences from fully sequenced genomes of *A. thaliana*^100^ and *E. grandis*^29^, to classify the putative genes found in *P. cattleyanum* according to the previous classification in the subfamilies TPS-a,-b,-c,-e/f, and -g by sequence similarity^26^.

To examine the evolutionary history of TPS genes, a second analysis including more species (*E. grandis*, *E. globulus*, *A. thaliana*, *P. trichocarpa*, *V. vinifera*, *C. citriodora*, and *M. alternifolia*) was carried out. We generated a tree with TPS sequences related to primary metabolism (subfamilies -c, -e, and -f) with a total of 45 sequences and a second tree related to secondary metabolism (subfamilies a, b, g) including 360 sequences^29,32,55^.

The functionally characterized pinene (RtTPS1 and RtTPS2 accession number AXY92166 and AXY92167, respectively) and caryophyllene synthases (RtTPS3 and RtTPS4 accession numbers AXY92168 and AXY92169) from *Rhodomyrtus tomentosa*^52^, pinene synthase (EpTPS1 accession number MK873024) and 1,8-cineole synthases (EpTPS2 and EpTPS3 accession numbers MK873025 and QCQ05478) from *Eucalyptus polybractea*^56^, beta cayophyllene synthase (Eucgr. J01451) from *E. grandis*^29^, myrcene synthase from *Antirrhium majus* (AAO41727)^101^, two isoprene synthase genes from *E. globulus* (EglobTPS106), *E. grandis* (Eucgr. K00881)^29^ and five linalool synthases from *Oenothera californica* (AAD19841)^63^, *Clarkia breweri* (AAD19840), *Clarkia concinna* (AAD19839), and *Fragaria x ananassa* (CAD57106)^102^ were also included in the phylogenetic analysis to assess the homology of known TPS to *Psidium* genes.

For each dataset used to construct the trees, we first aligned the amino acid sequences of putative TPS genes using ClustalW implemented within MEGA v7.0 software package^103^. Due to high levels of variation and variable exon counts between taxa, we trimmed the alignment using Gblocks^104^ with the following parameters: smaller final blocks, gap positions within the final blocks, and less strict flanking positions. We used the maximum-likelihood method implemented in PhyML v2.4.4^105^ online web server^106^ to perform the phylogenetic analysis. The JTT + G + F was the best-fit substitution model selected with ModelGenerator for protein analyses^107^. The confidence values in the tree topology were assessed by running 100 bootstrap replicates. Trees were visualized using Figtree v1.4.4^108^.

### Molecular evolutionary analysis involving TPS-b

To understand the molecular evolution at the amino acid level and the intensity of natural selection acting on metabolism in a specific clade, we used a tree based on codon alignment produced by the maximum-likelihood method using the software EasyCodeML^109^. We retrieved Coding Sequencing (CDS) sequences from TPS-b genes from *A. thaliana*, *E. grandis, P. cattleyanum, V. vinifera* and *P. trichocarpa* species in Phytozome v11 (http://phytozome.jgi.doe.gov/; last accessed November 2020), to use in positive selection analysis. The dataset included 76 sequences and 389 amino acids from five species. We performed statistical analysis using the CodeML program in PAML version 4.9 software using the site, branch, and branch-site models^110^, implemented in EasyCodeML^109^.

Parameter estimates (ω) and likelihood scores^111^ were calculated for the three pairs of models. These were M0 (one-ratio, assuming a constant ω ratio for all coding sites) vs. M3 (discrete, allowed for three discrete classes of ω within the gene), M1a (nearly neutral, allowed for two classes of ω sites: negative sites with ω0 < 1 estimated from our data and neutral sites with ω1 = 1) vs. M2a (positive selection, added a third class with ω2 possibly > 1 estimated from our data), and M7 (beta, a null model in which ω was assumed to be beta-distributed among sites) vs. M8 (beta and ω, an alternative selection model that allowed an extra category of positively selected sites)^112^.

A series of branch models and branch site models were tested: the one-ratio model for all lineages and the two-ratio model, where the original enzyme functional evolution occurred. The branch-site model assumes that the branches in the phylogeny are divided into the foreground (the one of interest for which positive selection is expected) and background (those not expected to exhibit positive selection).

Likelihood ratio tests (LRT) were conducted to determine which model measured the statistical significance of the data. The twice the log likelihood difference between each pair of models (2ΔL) follows a chi-square distribution with the number of degrees of freedom equal to the difference in the number of free parameters, resulting in a p-value for this^113^. A significantly higher likelihood of the alternative model compared to the null model suggests positive selection. Positive sites with high posterior probabilities (> 0.95) were obtained using empirical Bayes analysis. If ω > 1, then there is a positive selection on some branches or sites, but the positive selection sites may occur in very short episodes or on only a few sites during the evolution of duplicated genes; ω < 1 suggests a purifying selection (selective constraints), and ω = 1 indicates neutral evolution. Finally, naive empirical Bayes (NEB) approaches were used to calculate the posterior probabilities that a site comes from the site class with ω > 1^112^. The selected sites and images of protein topology were predicted using Protter^114^.

### Transcriptome analysis

For expression analysis, we used the published RNA-Seq dataset from leaves for the yellow and red morphotypes of *P. cattleyanum*^115^. To verify the quality of reads and the presence of Illumina adaptors, we used the FastQC software (http://www.bioinformatics.babraham.ac.uk/projects/fastqc/). Based on these data, we used the Trim Galore software (http://www.bioinformatics.babraham.ac.uk/projects/trim_galore/) to eliminate read strings with a quality below 30 and adapter sequences.

Two replicates from the red morphotype and two from the yellow morphotype, corresponding to four RNAseq libraries, were aligned on the draft genome assembly of each morphotype (unpublished data) using TopHat2^116^. The read count tables mapped to each gene were generated using the featureCounts module of the Subread software^117^, from the bam anchor files generated by TopHat2. The criteria used to create the counting tables were as follows: fragments (pairs of reads) were counted instead of individual reads, pairs of reads anchored on different chromosomes or anchoring on identical chromosomes but on different strands were not considered, and neither were the reads anchored in multiple places in the genome.

We used the DESeq2 package version 1.36^118^ to perform statistical analysis and identify differential expression. We analyzed the counting tables using a false discovery rate (FDR) of 0.05, log2 fold change ≥  ± 1^119^ and separated them into a group formed by the “up-regulated” genes and another formed by the “down-regulated” genes.

As the genome of each morphotype was assembled separately and corresponded to the same evaluated species in question, we performed two independent comparative transcriptomic analyses: a comparison of morphotype red leaf against yellow leaf anchoring in red morphotype genome (i) and in yellow genome (ii). We evaluated the differential expression considering each gene found in each morphotype, and were able to detect more genes under differential gene expression (DGE), considering that some gene copies were detected only in one of the reference genomes.

### Ethical standards

The yellow and red morphotypes of *Psidium cattleyanum* were sampled originally as part of the project “Genomics and Transcriptomics Analysis of *Psidium cattleyanum* Sabine (Myrtaceae)”. The studied samples were collected in full compliance with specific federal permits issued by the approved by the Brazilian Ministry of Environment (MMA) and the Chico Mendes Institute for Biodiversity Conservation (ICMBio), and approved by the Biodiversity Information and Authorization System (SISBIO 43338-2) and National System for Governance of Genetic Heritage and Associated Traditional Knowledge (SisGen A7B0331). The studied plants are kept in an ex situ collection at the Federal University of Rio Grande do Sul (UFRGS). Exsiccates will be deposited in the ICN herbarium of UFRGS. As official authorities in Brazil reported, the species used in this study are not endangered or protected in the Rio Grande do Sul State, where the sampling occurred.

## Supplementary Information

Supplementary Figures.

Supplementary Tables.

## Data Availability

All data generated or analysed during this study are included in this published article and its supplementary information files.
