# Supplementary material for: Genome-wide identification, expression profile and evolutionary relationships of TPS genes in the neotropical fruit tree species Psidium cattleyanum
Source: Sci Rep. 2023 Mar 9;13:3930. doi: 10.1038/s41598-023-31061-5 (PMC9998390; doi:10.1038/s41598-023-31061-5)
Supplement: Supplementary file 1 — Supplementary Figures. [file 41598_2023_31061_MOESM1_ESM.pdf]

Genome-wide study of the terpene synthase gene family in *Psidium cattleyanum* S. reveals expansion and positive selection of TPS-b genes acting on monoterpene synthesis

Drielli Canal1, Frank Lino Guzman Escudero2, Luiza Alves Mendes3, Marcia Flores da Silva Ferreira4 & Andreia Carina Turchetto-Zolet1*

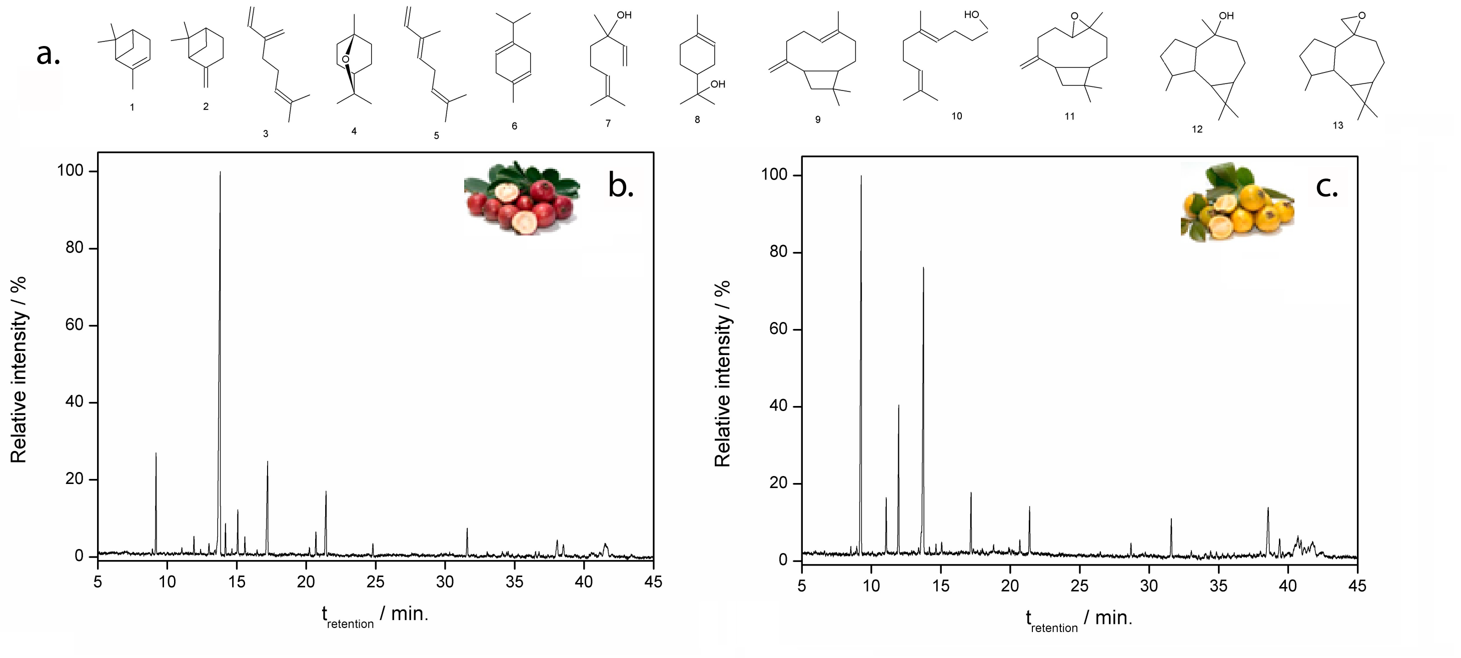

Supplementar Figure S1. Chromatogram of *Psidium cattleyanum* essential oil. a. Red and b. Yellow morphotypes. c. Chemical structures of the compounds identified in the essential oil: 1) α-pinene; 2) β-pinene; 3) β-myrcene; 4) 1,8-cineole; 5) β-ocimene; 6) γ- terpinene; 7) linalool; 8) α-terpineol; 9) β-caryophyllene; 10) nerolidol; 11) caryophyllene oxide; 12) viridiflorol; 13) aromadendrene epoxide.

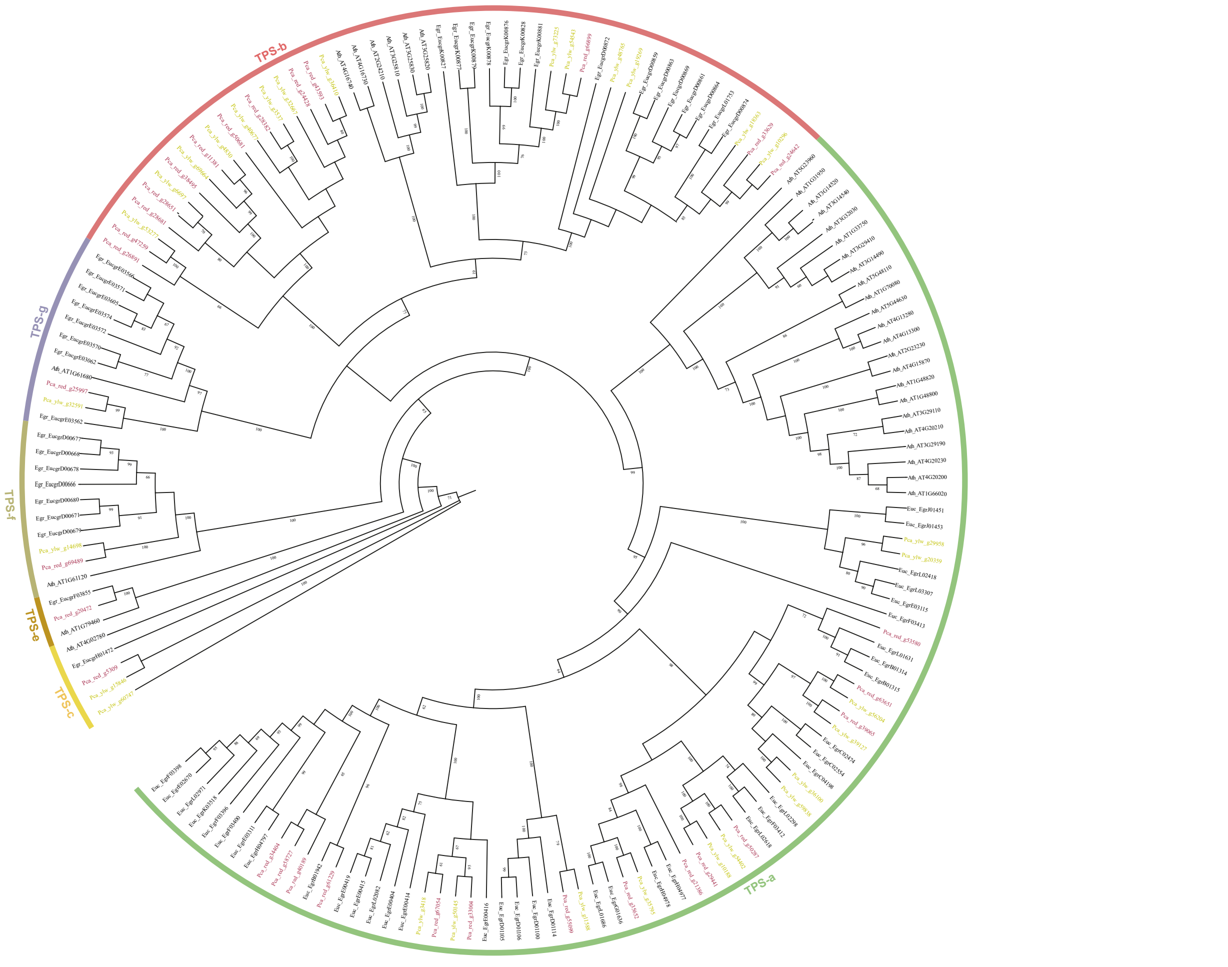

Supplementar Figure S2. Phylogenetic analysis of *Eucalyptus grandis*, *Arabdopisis thaliana* and *Psidium cattleianum* full length terpene synthase (TPS) enzymes using 164 sequences. The clusters correspond to TPS subfamilies: TPS-a, TPS-b, TPS-c, TPS-e/f and TPS-g. Protein alignments were conducted using the ClustalW algorithm and trees were inferred using maximum likelihood methods. The support values associated with the branches are bootstrapping, with values over 60% shown.
